# Differential BMP Signaling Mediates the Interplay Between Genetics and Leaflet Numbers in Aortic Valve Calcification

**DOI:** 10.1016/j.jacbts.2021.12.006

**Published:** 2022-03-23

**Authors:** Jae-Joon Jung, Azmi A. Ahmad, Saranya Rajendran, Linyan Wei, Jiasheng Zhang, Jakub Toczek, Lei Nie, Gunjan Kukreja, Mani Salarian, Kiran Gona, Mean Ghim, Raja Chakraborty, Kathleen A. Martin, George Tellides, Donald Heistad, Mehran M. Sadeghi

**Affiliations:** a Section of Cardiovascular Medicine and Cardiovascular Research Center, Yale University School of Medicine, New Haven, Connecticut, USA; b VA Connecticut Healthcare System, West Haven, Connecticut, USA; c Department of Surgery, Yale University School of Medicine, New Haven, Connecticut, USA; d Division of Cardiovascular Medicine, University of Iowa Carver College of Medicine, Iowa City, Iowa, USA

**Keywords:** aortic stenosis, aortic valve, bicuspid aortic valve, calcification, mouse models, BAV, bicuspid aortic valve, BMP, bone morphogenic protein, CAVD, calcific aortic valve disease, DCBLD2, discoidin, CUB and LCCL domain containing 2, EC, endothelial cell, pVIC, porcine valvular interstitial cell, RT-PCR, reverse-transcription polymerase chain reaction, SMAD, homolog of Caenorhabditis elegans Sma and the Drosophila mad, mothers against decapentaplegic, siRNA, small interfering RNA, TAV, tricuspid aortic valve, VIC, valvular interstitial cell, WT, wild type

## Abstract

Expression of a neuropilin-like protein, DCBLD2, is reduced in human calcific aortic valve disease (CAVD). DCBLD2-deficient mice develop bicuspid aortic valve (BAV) and CAVD, which is more severe in BAV mice compared with tricuspid littermates. In vivo and in vitro studies link this observation to up-regulated bone morphogenic protein (BMP)2 expression in the presence of DCBLD2 down-regulation, and enhanced BMP2 signaling in BAV, indicating that a combination of genetics and BAV promotes aortic valve calcification and stenosis. This pathway may be a therapeutic target to prevent CAVD progression in BAV.

## Highlights

• The neuropilin-like protein, DCBLD2, is down-regulated in aortic valves of patients undergoing valve replacement for aortic stenosis.
• About 50% of DCBLD2-deficient mice develop bicuspid aortic valve. These animals have a high prevalence of calcific aortic valve disease with typical features of human disease, including aortic stenosis.
• DCBLD2 down-regulation up-regulates bone morphogenic protein 2, a key mediator of calcification. Despite a similar level of bone morphogenic protein 2 between bicuspid and tricuspid aortic valves, downstream signaling and expression of the calcification marker, osteocalcin are more pronounced in bicuspid aortic valves.
• Introducing a clinically relevant model of calcific aortic valve disease, these findings may explain how a combination of genetic background and bicuspid aortic valve promotes aortic valve calcification and stenosis.

## Visual Abstract

**Figure:**
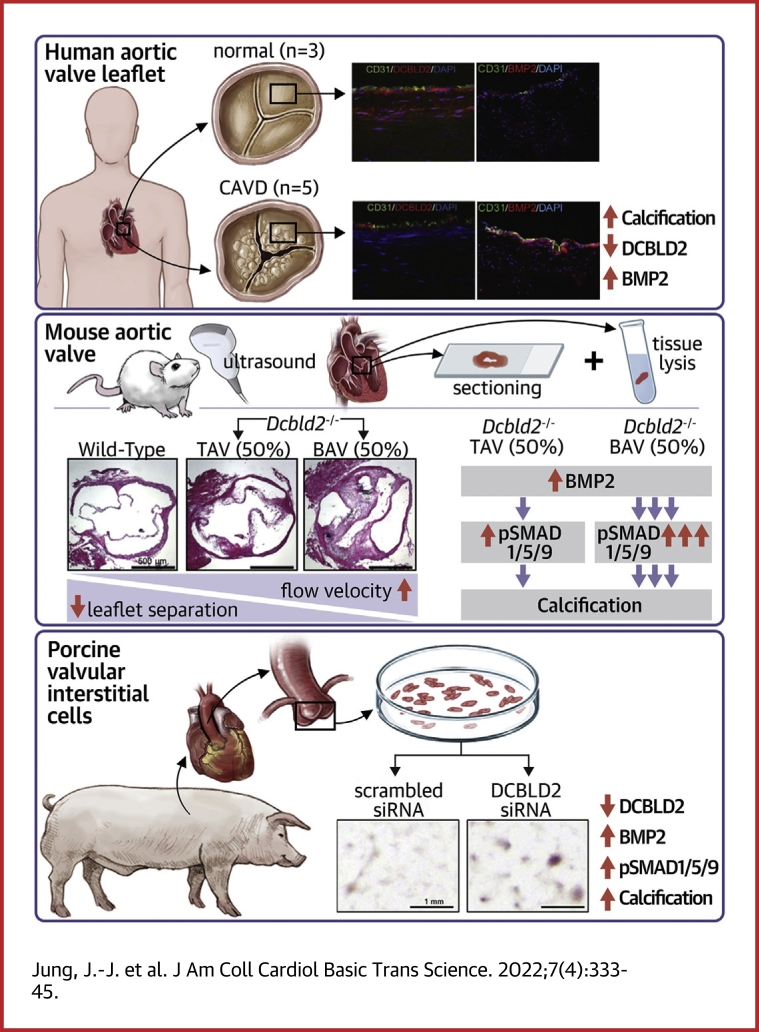

Calcific aortic valve disease (CAVD) is the most common cause of aortic stenosis. Leaflet thickening and fibrosis, calcification, and hemodynamically significant stenosis are the hallmarks of the disease, which often takes decades to develop.^1^ There is currently no medical therapy to prevent or reverse CAVD.^2^ The absence of appropriate animal models that mimic human CAVD is a major limitation to understanding CAVD pathophysiology and the development of effective medical therapy.^3^ Bicuspid aortic valve (BAV) is a major risk factor for CAVD, and patients with BAV have earlier, more aggressive disease progression compared with tricuspid aortic valve (TAV). Interestingly, relatives of patients with familial BAV who have a TAV are at increased risk for developing CAVD, pointing to the presence of common factor(s) that predispose to BAV development and CAVD in both BAV and TAV.^4^^,^^5^

The pathogenesis of CAVD is complex and involves the differentiation of quiescent fibroblast-like valvular interstitial cells (VICs) to myofibroblast and osteoblast-like interstitial cells. Like in bone mineralization, bone morphogenic protein (BMP), and more specifically, BMP2 signaling is implicated in valvular calcification. However, existing evidence indicates that BMP2 is insufficient by itself and additional factors are required to induce aortic valve calcification.^6^ It is possible that local changes in blood flow in BAV leads to biomechanical alterations that contribute to the accelerated progression of CAVD. However, in the absence of representative animal models, the molecular mechanisms of the interplay between the leaflet numbers and genetic factors that predispose to CAVD remain unclear.^3^

Discoidin, CUB and LCCL Domain Containing 2 (DCBLD2, also known as endothelial and smooth muscle cell-derived neuropilin-like protein [ESDN]) is a transmembrane protein^7^^,^^8^ implicated in the regulation of growth factor signaling, vascular remodeling, and angiogenesis.^9^, ^10^, ^11^, ^12^, ^13^ A recent human aortic valve gene profiling study suggested that *DCBLD2* transcripts are reduced in stenotic aortic valves.^14^^,^^15^ In the course of our studies aimed at evaluating the role of DCBLD2 in vascular remodeling, we noticed the presence of BAV with thickened leaflets in a subset of *Dcbld2*^*−/−*^ mice. This led us to investigate the potential role of DCBLD2 in aortic valve pathology and to take advantage of this model to address how the interplay between the genetic background and leaflet numbers determines aortic valve calcification and stenosis. Here, we show that DCBLD2 expression is reduced in human CAVD, and *Dcbld2*^*−/−*^ mice develop BAV and CAVD with typical features of human disease. In vitro and in vivo studies link the more prominent valvular calcification of BAV to BMP2 signaling, which despite similar levels of *Bmp2* expression between bicuspid and tricuspid valves, is enhanced in BAV.

## Methods

Detailed materials and methods are found in the Supplemental Appendix.

### Human tissues

Normal human aortic valves were obtained from deceased organ donors, and aortic valves with advanced CAVD were obtained from patients undergoing aortic valve replacement for symptomatic aortic stenosis under protocols approved by Yale Institutional Review Board.

### Animal models

The generation of *Dcbld2*^−/−^ mice on a C57BL/6 background and endothelial-specific conditional knockout mice (*Cdh5-Cre*/*Dcbld2*^fl/−^) were reported previously.^10^ Animals of both sexes were used for these studies. All animal procedures were performed in accordance with protocols approved by Yale University and Veterans Affairs Connecticut Healthcare System institutional animal care and use committees.

### Statistical analysis

Values are expressed as either the mean ± SD (for normally distributed data) or median with 25th and 75th percentiles (IQR) for nonparametric data. Normally distributed data were compared using 2-tailed *t-test*, paired Student's *t*-test, or 1-way analysis of variance with post hoc Tukey's method for multiple pairwise comparisons (>2 groups). The data that did not pass the normality test were compared using the Mann-Whitney *U* test or Kruskal-Wallis test (for >2 groups). Chi-square analysis was used to compare the counts of different categories between 2 independent groups. A *P* value <0.05 was considered significant. All statistical analyses were performed using GraphPad Prism 9 (GraphPad Software).

## Results

### DCBLD2 expression is reduced in human CAVD

**Figure 1:**
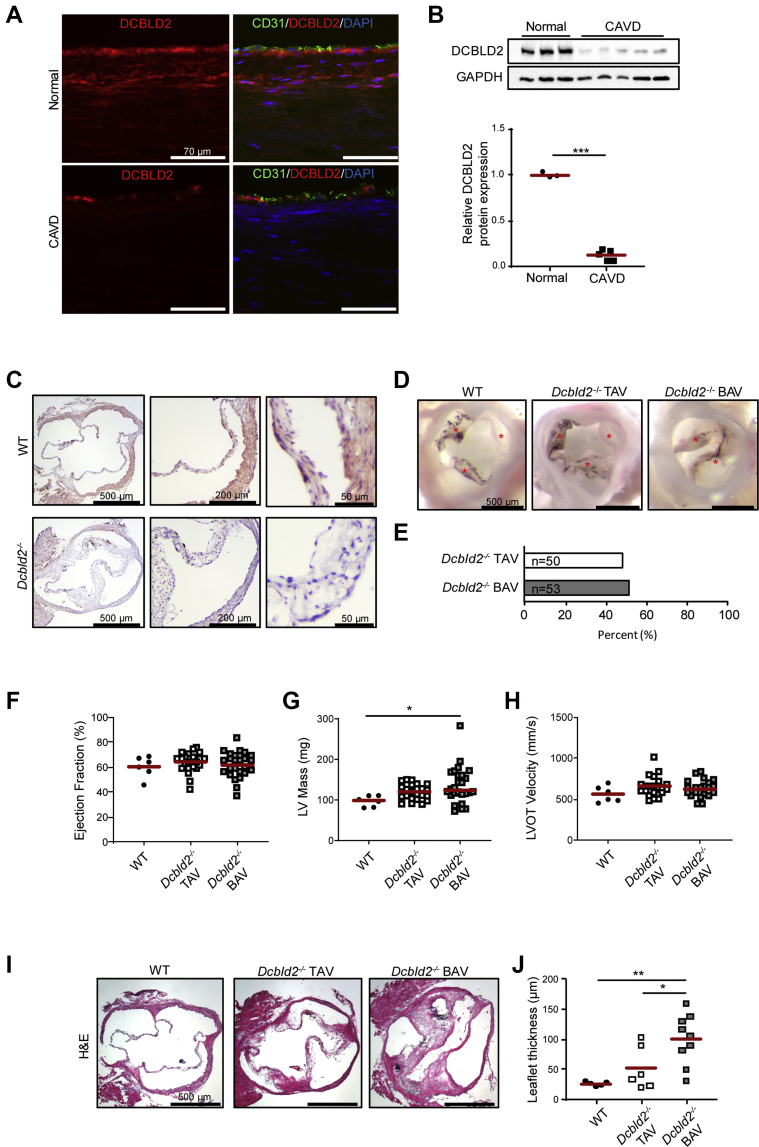
DCBLD2 Is Down-Regulated in Human CAVD, and Dcbld2 Deletion Leads to Isolated BAV and Valvular Remodeling in a Subset of Mice **(A)** Immunofluorescent staining of DCBLD2 **(red)** and CD31 **(green)** in normal human aortic valve leaflets **(upper panels)** and aortic valve leaflets with advanced CAVD **(lower panels)**. Nuclei are stained **blue** with DAPI. **(B)** Western blot analysis **(upper panel)** and quantification **(lower panel)** of DCBLD2 expression in normal human aortic valve leaflets and aortic valve leaflets with advanced CAVD. ∗∗∗*P <* 0.001 (2-tailed *t-test*). **(C)** Immunohistochemical staining of DCBLD2 in WT **(upper panels)** and *Dcbld2*^−/−^**(lower panels)** murine aortic valves. **(D)** Photographic images of 9- to 12-month-old WT **(left)**, *Dcbld2*^−/−^ TAV **(middle)**, and *Dcbld2*^−/−^ BAV **(right)** aortic valves. Individual leaflets are marked with **asterisks**. **(E)** Prevalence of TAV and BAV in *Dcbld2*^−/−^ mice. **(F-H)** LV ejection fraction **(F)**, LV mass **(G)**, and LV outflow tract velocity **(H)** assessed by echocardiography in WT, *Dcbld2*^−/−^ TAV, and *Dcbld2*^−/−^ BAV mice. ∗*P <* 0.05 (Kruskal-Wallis test with Dunn’s multiple comparisons). **(I)** Hematoxylin and eosin staining of WT **(left)**, *Dcbld2*^−/−^ tricuspid **(middle)**, and *Dcbld2*^−/−^ bicuspid **(right)** aortic valves. **(J)** Leaflet thickness of WT aortic valve, *Dcbld2*^−/−^ TAV, and *Dcbld2*^−/−^ BAV. ∗*P <* 0.05, ∗∗*P <* 0.01 (1-way analysis of variance with Tukey’s multiple comparisons). BAV = bicuspid aortic valve; CAVD = calcific aortic valve disease; LV = left ventricular; TAV = tricuspid aortic valve; WT = wild-type.

We evaluated DCBLD2 expression by immunostaining in normal human aortic valves obtained from deceased organ donors, and aortic valves with advanced CAVD obtained from patients undergoing aortic valve replacement for symptomatic aortic stenosis (Figure 1A, Supplemental Figure 1A). In normal aortic valves, DCBLD2 is expressed in CD31-positive endothelial cells (ECs) that cover the leaflets, as well as the rest of the leaflets, which predominantly contains VIC. In contradistinction to normal valves, DCBLD2 expression is markedly diminished in aortic valve leaflets with advanced CAVD. Quantitative analysis of DCBLD2 protein by Western blotting (Figure 1B) confirmed the significant reduction of DCBLD2 protein expression in CAVD, which is associated with a significant reduction in *DCBLD2* mRNA expression, as detected by reverse-transcription polymerase chain reaction (RT-PCR) (Supplemental Figure 1B).

### BAV, CAVD, and aortic stenosis in *Dcbld2*^−/−^ mice

To explore the role of DCBLD2 in aortic valve pathobiology, we first evaluated its expression in wild-type (WT) and *Dcbld2*^−/−^ murine aortic valves. Like in human aortic valves, in the aortic valves of WT mice, DCBLD2 was detectable by immunostaining throughout the leaflet, including CD31-positive ECs (Figure 1C, Supplemental Figure 1C). As expected, *Dcbld2*^−/−^ aortic valves did not express any DCBLD2 (Figure 1C). Importantly, about 50% of *Dcbld2*^−/−^ mice of both sexes were noted to have BAV (Figures 1D and 1E). The presence of BAV was not associated with any unrelated structural cardiac abnormality. Echocardiography in 9- to 12-month-old animals showed no difference in left ventricular ejection fraction or outflow tract velocity between *Dcbld2*^*−/−*^ BAV, *Dcbld2*^*−/−*^ TAV, and WT mice. However, the left ventricular mass was significantly higher in *Dcbld2*^*−/−*^ BAV mice compared with WT animals (WT: 98.89 [IQR: 81.48-110.7] mg; *Dcbld2*^*−/−*^ TAV: 120.2 [IQR: 103.7-137.2] mg; and *Dcbld2*^*−/−*^ BAV: 124 [IQR: 113.5-163.1] mg, n = 6 to 26; *P <* 0.05 for *Dcbld2*^−/−^ BAV vs WT) (Figures 1F to 1H).

**Figure 2:**
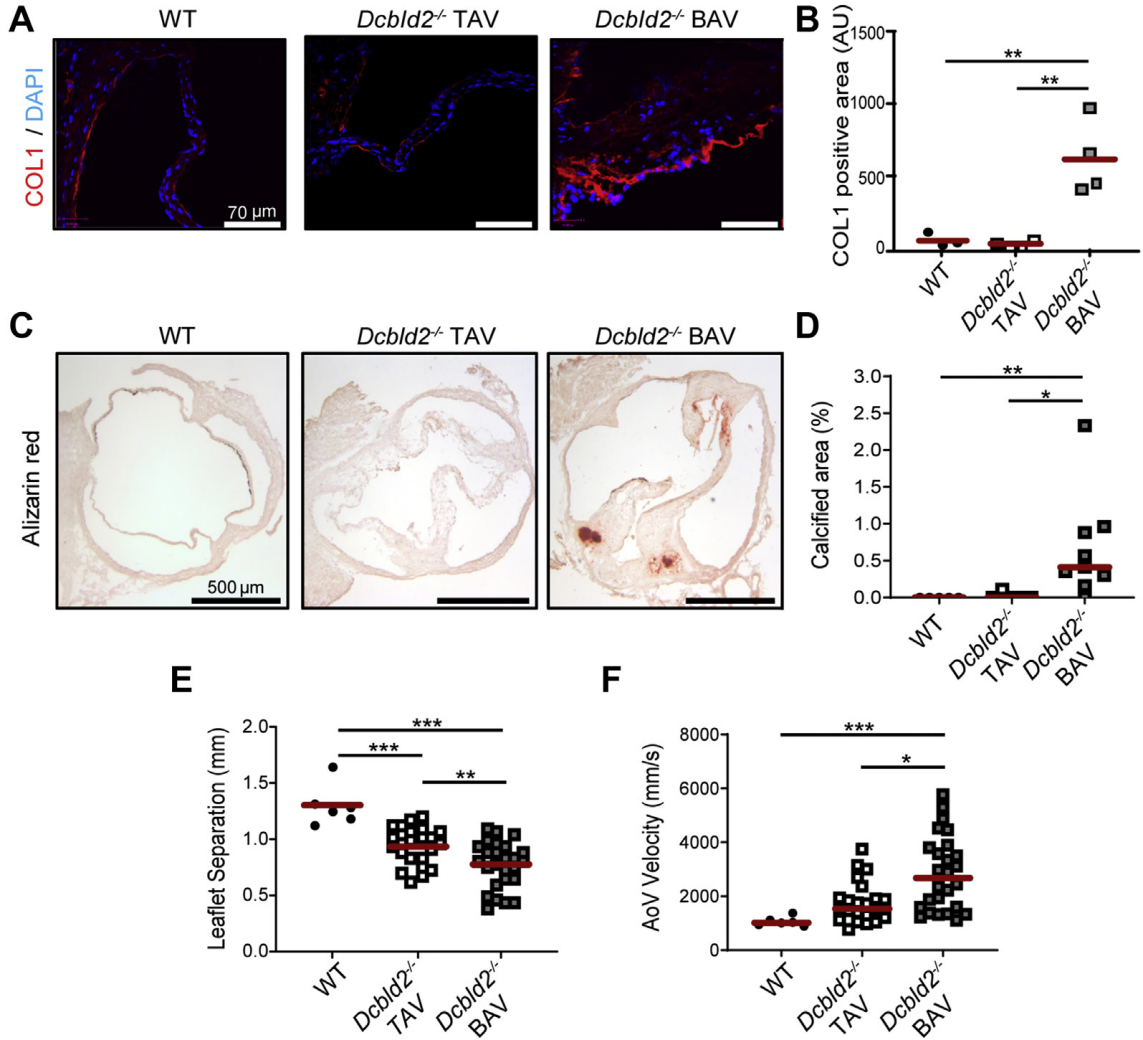
Dcbld2 Deficiency Promotes Valvular Fibrosis, Calcification, and Stenosis **(A and B)** Immunofluorescent staining **(A)** and quantification **(B)** of collagen type I (COL1, **red**) in WT aortic valve **(left)**, *Dcbld2*^−/−^ TAV **(middle)**, and *Dcbld2*^−/−^ BAV **(right)** leaflets. ∗∗*P <* 0.01 (1-way analysis of variance with Tukey’s multiple comparisons). **(C and D)** Alizarin red staining **(C)** and quantification **(D)** of valvular calcification in WT **(left)**, *Dcbld2*^−/−^ TAV **(middle)**, and *Dcbld2*^−/−^ BAV **(right)** mice. ∗*P <* 0.05, ∗∗*P <* 0.01 (Kruskal-Wallis test with Dunn’s multiple comparisons). **(E and F)** Aortic valve leaflet separation **(E)** and peak transvalvular velocity **(F)** in WT, *Dcbld2*^−/−^ TAV, and *Dcbld2*^−/−^ BAV mice assessed by echocardiography. ∗*P <* 0.05, ∗∗*P <* 0.01, ∗∗∗*P <* 0.001 (1-way analysis of variance with Tukey’s multiple comparisons for **E**, and Kruskal-Wallis test with Dunn's multiple comparisons for **F**). Abbreviations as in Figure 1.

To investigate the development of CAVD in *Dcbld2*^−/−^ mice, we assessed the key features of CAVD, namely, valvular fibrosis and calcification in 9- to 12-month-old mice in comparison with age-matched WT animals. *Dcbld2*^−/−^ BAV leaflets were significantly thicker than WT and *Dcbld2*^−/−^ TAV leaflets (WT: 26.5 ± 3.8 μm; *Dcbld2*^−/−^ TAV: 34.3 ± 12.1 μm; *Dcbld2*^−/−^ BAV: 100.6 ± 41.6 μm, n = 5 to 9; *P <* 0.01 for BAV vs WT and *P <* 0.05 for BAV vs TAV) (Figures 1I and 1J). Interestingly, not all BAV leaflets were thickened, and a small subset of animals with TAV had leaflet thickening (Figure 1J). Masson’s trichrome staining suggested that fibrosis contributes to leaflet thickening in *Dcbld2*^−/−^ mice (Supplemental Figure 2). Indeed, the collagen type I content, as detected by immunostaining, was significantly higher in *Dcbld2*^−/−^ BAV compared with WT leaflets (Figures 2A and 2B). The *Dcbld2*^−/−^ TAV leaflets without thickening contained less collagen with a patchy distribution.

Alizarin red staining of aortic valves showed multiple calcified nodules in *Dcbld2*^−/−^ BAV leaflets, especially in the hinge area similar to human CAVD, that spared the aortic sinus. No calcification was detected in WT aortic valves. Quantification of Alizarin red staining showed significantly higher calcification in *Dcbld2*^−/−^ BAV compared with *Dcbld2*^−/−^ TAV and WT valve leaflets, as % of the leaflet area (WT: 0% [IQR: 0%-0%]; *Dcbld2*^−/−^ TAV: 0% [IQR: 0%-0.06%]; *Dcbld2*^−/−^ BAV: 0.41 [IQR: 0.23%-0.92%], n = 5 to 9; *P <* 0.01 for BAV vs WT and *P <* 0.05 for BAV vs TAV) (Figures 2C and 2D). Of note, a subset of BAV had no calcification. In addition, a subset of *Dcbld2*^−/−^ TAV showed thickened leaflets with small, calcified nodules suggesting that the effect of DCBLD2 deficiency is not confined to BAV (Figures 1I, 1J, 2C, and 2D). Finally, no calcification was detectable in 3-month-old animals (not shown), indicating that this calcification is an acquired phenotype.

We assessed the physiological significance of CAVD in *Dcbld2*^−/−^ mice of both sexes by echocardiography (Supplemental Videos 1 and 2, Supplemental Figure 3). Compared with WT mice, both *Dcbld2*^−/−^ TAV and BAV mice showed significantly reduced leaflet separation, and the leaflet separation was significantly lower in *Dcbld2*^−/−^ BAV than TAV mice (WT: 1.29 ± 0.18 mm; *Dcbld2*^−/−^ TAV: 0.94 ± 0.17 mm; *Dcbld2*^−/−^ BAV: 0.76 ± 0.21 mm, n = 6 to 26; *P <* 0.001 for *Dcbld2*^−/−^ TAV vs WT; *P <* 0.001 for *Dcbld2*^−/−^ BAV vs WT; and *P <* 0.01 for *Dcbld2*^−/−^ BAV vs *Dcbld2*^−/−^ TAV) (Figure 2E). The reduction in leaflet separation in *Dcbld2*^−/−^ BAV mice was associated with a significant increase in aortic valve peak flow velocity relative to both WT and *Dcbld2*^−/−^ TAV mice (WT: 1,019 [IQR: 931-1,180] mm/s; *Dcbld2*^−/−^ TAV: 1,535 [IQR: 1,212-1,946] mm/s; *Dcbld2*^−/−^ BAV: 2,679 [IQR: 1,533-3,825] mm/s; *P <* 0.001 for *Dcbld2*^−/−^ BAV vs WT; and *P <* 0.05 for *Dcbld2*^*−/−*^ BAV vs *Dcbld2*^*−/−*^ TAV) (Figure 2F). Defining severe aortic stenosis as a maximum leaflet separation less than the mean minus 2.5 SD of leaflet separation in WT mice (0.83 mm)^16^ or a peak transvalvular flow velocity >2,400 mm/s, the incidence of severe aortic stenosis in BAV was more than twice the incidence in TAV *Dcbld2*^−/−^ mice (leaflet separation: 26% for TAV and 58% for BAV; *P <* 0.05; flow velocity: 17% for TAV and 54% for BAV; *P <* 0.01). Of the 18 animals with highly elevated peak aortic valve velocities, 4 (3 TAV, 1 BAV) also had mild aortic regurgitation by color Doppler imaging. Combined, these data indicate that *Dcbld2*^−/−^ mice can develop CAVD with hemodynamically significant aortic stenosis, and the disease is more severe in animals with BAV.

### DCBLD2 regulates BMP2 expression and signaling in valvular cells

**Figure 3:**
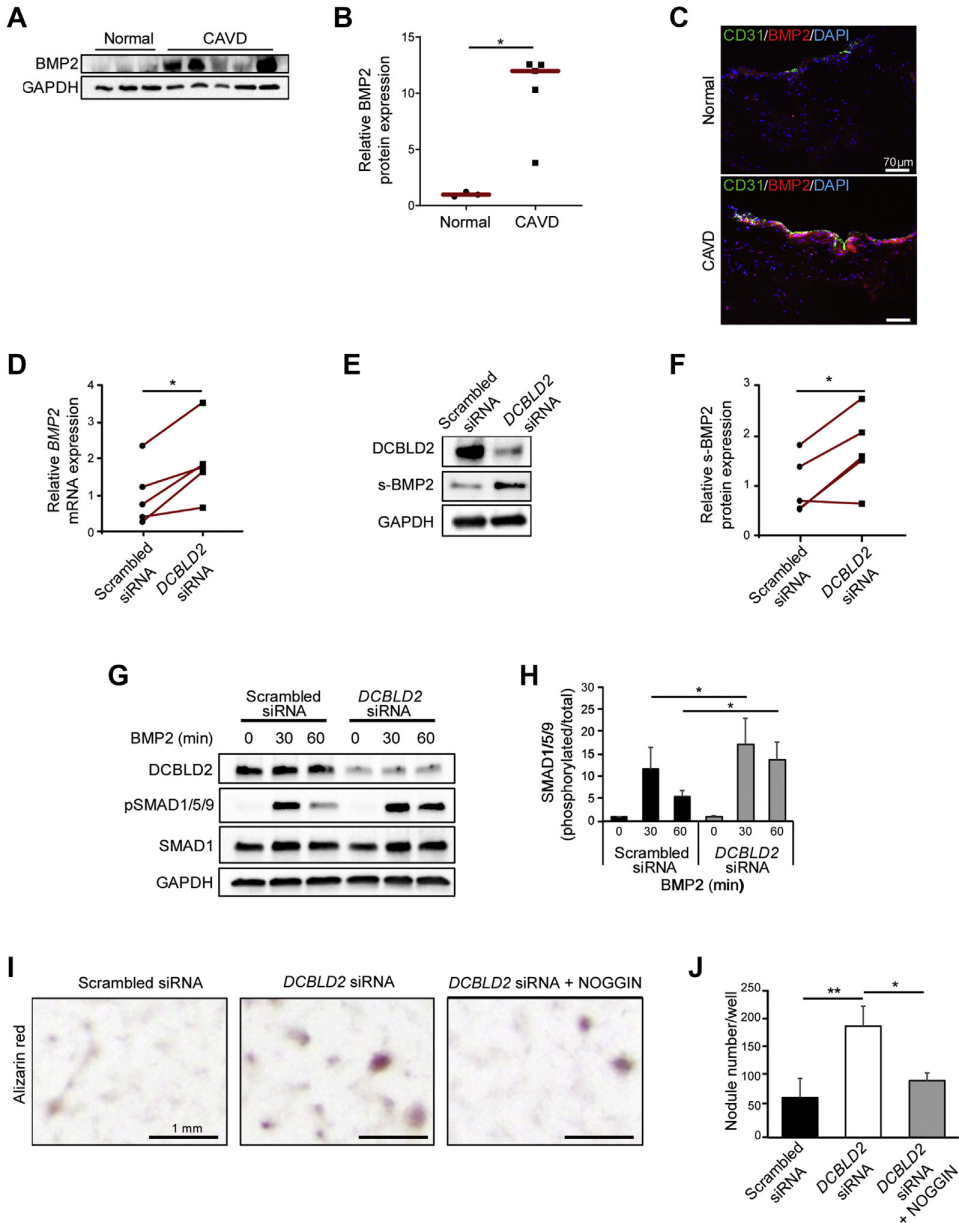
DCBLD2 Regulates BMP2 Expression and Signaling in VICs **(A and B)** Western blot analysis **(A)** and quantification **(B)** of BMP2 expression in normal human aortic valve leaflets and aortic valve leaflets with advanced CAVD. ∗*P <* 0.05 (Mann-Whitney *U* test). **(C)** Immunofluorescent staining of BMP2 **(red)** and CD31 **(green)** in normal human aortic valve leaflets **(upper panel)** and aortic valve leaflets with advanced CAVD **(lower panel)**. Nuclei are stained **blue** with DAPI. **(D-F)** RT-PCR analysis of *Bmp2* mRNA **(D)**, and Western blot analysis of s-BMP2 protein expression **(E)** and its quantification **(F)** in pVIC transfected with scrambled or *DCBLD2* siRNAs. ∗*P <* 0.05 (paired Student's *t*-test). **(G and H)** Western blot analysis **(G)** and its quantification **(H)** of SMAD1/5/9 phosphorylation in pVIC treated with porcine BMP2 (50 ng/mL) for the indicated time points. ∗*P <* 0.05 (paired Student's *t*-test). **(I and J)** Representative images **(I)** and quantification **(J)** of Alizarin red staining of pVIC transfected with scrambled or DCBLD2 siRNAs, or DCBLD2 siRNA with recombinant human Noggin. n = 3. ∗*P <* 0.05, ∗∗*P <* 0.01 (1-way analysis of variance with Tukey’s multiple comparisons). The Figure is representative of 2 independent experiments. CAVD = calcific aortic valve disease; pVIC = porcine valvular interstitial cell; RT-PCR = reverse-transcription polymerase chain reaction; s-BMP2 = secreted BMP2; siRNA = small interfering RNA; VIC = valvular interstitial cell.

Evaluation of BMP2 expression, as a key mediator of valvular calcification in CAVD,^6^ by Western blotting showed significantly higher protein levels in human aortic valves with advanced CAVD compared with normal aortic valves (*P <* 0.05) (Figures 3A and 3B). Immunostaining showed rare foci of BMP2 in normal aortic valves, which contrasted with the diffuse BMP2 expression in calcified aortic valves that pointed to both valvular EC and VIC as potential sources of BMP2 production (Figure 3C). Therefore, we evaluated the effect of *Dcbld2* down-regulation on BMP2 expression and signaling in VIC and EC. Given the difficulty isolating and culturing large numbers of murine and human valvular cells, porcine VIC (pVIC) are routinely used as a model to study the molecular mechanisms of valvular calcification in vitro.^17^ Small interfering RNA (siRNA)-mediated DCBLD2 down-regulation significantly increased *BMP2* mRNA expression as well as BMP2 protein secretion to culture media in pVIC (*P <* 0.05) (Figures 3D to 3F). Likewise, in human VIC short hairpin RNA-mediated DCBLD2 down-regulation increased BMP2 expression (*P <* 0.05) (Supplemental Figure 4). Interestingly, along with its effect on BMP2 production, siRNA-mediated DCBLD2 down-regulation significantly enhanced homolog of Caenorhabditis elegans Sma and the Drosophila mad, mothers against decapentaplegic (SMAD)1/5/9 phosphorylation in response to exogenous BMP2 in pVIC (Figures 3G and 3H). To evaluate the downstream effects of the changes in BMP2 expression and signaling, we assessed the effect of DCBLD2 down-regulation on pVIC calcification. As expected, pVIC cultured in osteogenic medium formed calcified nodules over a 3-day period. siRNA-mediated DCBLD2 down-regulation significantly enhanced nodule formation (scrambled siRNA: 68 ± 33 nodules/well; DCBLD2 siRNA: 189 ± 33 nodules/well; *P <* 0.01) (Figures 3I and 3J). Importantly, this effect was blocked in the presence of Noggin, a potent BMP inhibitor that prevents BMP binding to cell surface receptors,^18^ demonstrating that BMP is required for calcific nodule formation in the setting of DCBLD2 down-regulation (DCBLD2 siRNA: 189 ± 33 nodules/well; DCBLD2 siRNA with Noggin: 95 ± 13 nodules/well; *P <* 0.05) (Figures 3I and 3J).

**Figure 4:**
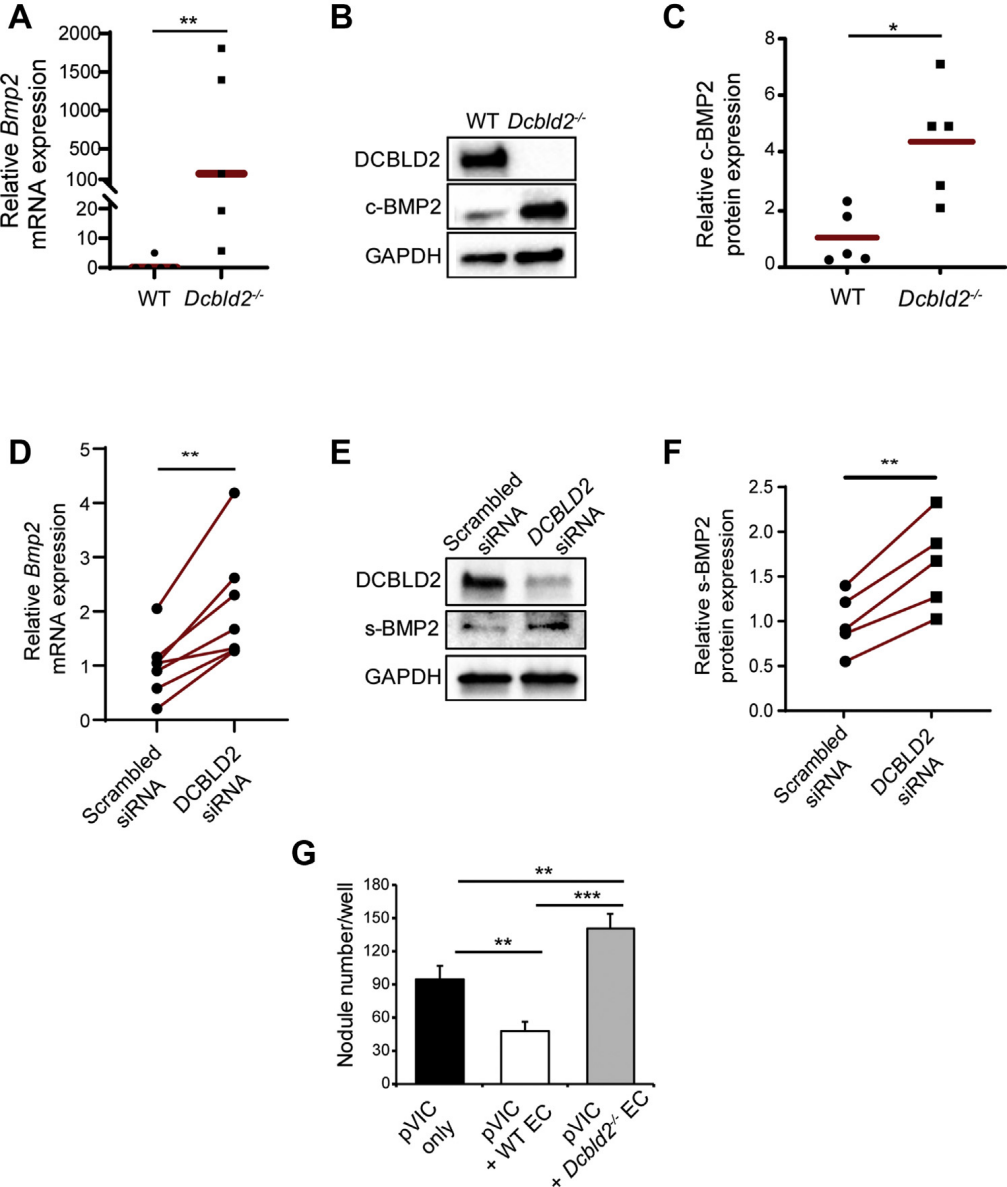
Endothelial DCBLD2 Regulates Endothelial BMP2 Expression and VIC Calcification **(A-C)** RT-PCR analysis of *Bmp2* mRNA **(A)**, and Western blot analysis of c-BMP2 protein expression **(B)** and its quantification **(C)** in WT and *Dcbld2*^−/−^ EC. ∗*P <* 0.05 (2-tailed *t-test*), ∗∗*P <* 0.01 (Mann-Whitney *U* test). **(D-F)** RT-PCR analysis of *Bmp2* mRNA **(D)** and Western blot analysis of s-BMP2 protein **(E)** expression and its quantification **(F)** in porcine valvular endothelial cells transfected with scrambled or DCBLD2 siRNAs. ∗∗*P <* 0.01 (paired Student's *t*-test). **(G)** Quantification of calcified nodule formation in pVIC alone and pVIC co-cultured with WT or *Dcbld2*^−/−^ EC. n = 3. ∗∗*P <* 0.01, ∗∗∗*P <* 0.001 (1-way analysis of variance with Tukey’s multiple comparisons). c-BMP2 = cytosolic BMP2; EC = endothelial cell; other abbreviations as in Figures 1 and 3.

Similar to the effect of DCBLD2 deficiency on BMP2 production by VIC, BMP2 mRNA and protein expression were markedly increased in *Dcbld2*^−/−^ ECs compared with WT cells (*P <* 0.01 and *P <* 0.05) (Figures 4A to 4C). The effect of DCBLD2 on BMP2 production was confirmed in porcine ECs where siRNA-mediated DCBLD2 down-regulation increased BMP2 mRNA expression and protein secretion (*P <* 0.01) (Figures 4D to 4F). Finally, although consistent with previous reports^19^ co-culture with WT murine EC significantly attenuated pVIC calcified nodule formation (pVIC only: 95 ± 12 nodules/well; pVIC with WT EC: 48 ± 8 nodules/well; *P <* 0.01) (Figure 4G), pVIC co-culture with *Dcbld2*^−/−^ ECs (which express higher levels of BMP2) had an opposite effect and significantly increased pVIC calcification (pVIC with *Dcbld2*^−/−^ EC: 140 ± 14 nodules/well; *P <* 0.01 vs pVIC only) (Figure 4G).

### Endothelial DCBLD2 deficiency is not sufficient for BAV and CAVD development

Cell culture studies indicated that DCBLD2 deficiency promotes a procalcific phenotype in ECs and VICs. To investigate the contribution of endothelial DCBLD2 deficiency to BAV and CAVD development, we compared *Cdh5-Cre*/*Dcbld2*^fl/−^ mice, where *Dcbld2* is constitutively deleted in ECs,^10^ with WT animals. Echocardiographic evaluation of the aortic valve in 1-year-old *Cdh5-Cre*/*Dcbld2*^fl/−^ mice showed no difference in leaflet separation and peak aortic valve velocity between these animals and WT mice (Supplemental Figure 5A and 5B). EC-specific DCBLD2 deficiency in aortic valve leaflets was confirmed by immunostaining in the *Cdh5-Cre*/*Dcbld2*^fl/−^ mice (Supplemental Figure 5C). None of the *Cdh5-Cre*/*Dcbld2*^fl/−^ mice had BAV (Supplemental Figure 5D), leaflet thickening (Supplemental Figure 5E), or calcification (not shown) on post-mortem analysis of the aortic valve. Combined, these data indicate that endothelial *Dcbld2* deficiency is not sufficient to promote BAV, leaflet thickening and calcification, or aortic stenosis.

### Differential BMP2 signaling in *Dcbld2*^−/−^ BAV and TAV

**Figure 5:**
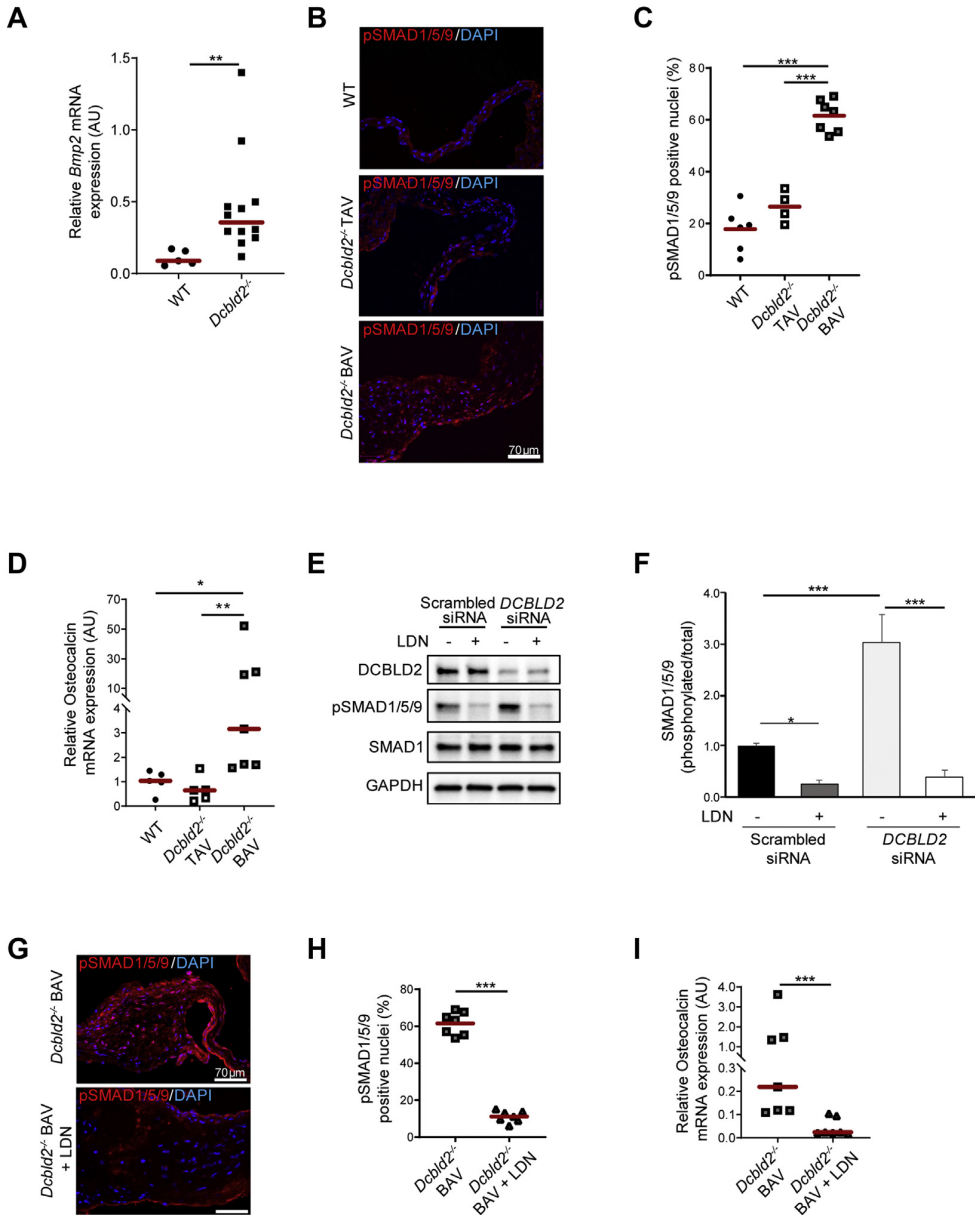
Differential BMP2 Signaling Underlies the Difference in Valvular Calcification Between Dcbld2^−/−^ BAV and TAV **(A)***Bmp2* mRNA expression in WT and *Dcbld2*^−/−^ aortic valve tissues. ∗∗*P <* 0.01 (Mann-Whitney *U* test). **(B and C)** Examples of phosphorylated SMAD1/5/9 immunofluorescent staining in **red (B)** and quantification **(C)** in WT aortic valve, *Dcbld2*^−/−^ TAV and *Dcbld2*^−/−^ BAV leaflets. Nuclei are stained **blue** with DAPI. ∗∗∗*P <* 0.001 (1-way analysis of variance with Tukey’s multiple comparisons). **(D)** Osteocalcin mRNA expression in WT, *Dcbld2*^−/−^ TAV, and *Dcbld2*^−/−^ BAV. ∗*P <* 0.05, ∗∗*P <* 0.01 (Kruskal-Wallis test with Dunn's multiple comparisons). **(E and F)** Western blot analysis **(E)** and quantification **(F)** of phosphorylated SMAD1/5/9 in pVIC transfected with scrambled or DCBLD2 siRNA in the presence or absence of LDN-193189 (0.5 μmol/L). ∗*P <* 0.05, ∗∗∗*P <* 0.001 (1-way analysis of variance with Tukey’s multiple comparisons). **(G and H)** Representative examples of phosphorylated SMAD1/5/9 immunofluorescent staining in **red (G)** and quantification **(H)** in *Dcbld2*^−/−^ BAV mice and *Dcbld2*^−/−^ BAV mice treated with LDN-193189 (6 mg/kg). Nuclei are stained **blue** with DAPI. ∗∗∗*P <* 0.001 (2-tailed *t-test*). **(I)** Osteocalcin mRNA expression in *Dcbld2*^−/−^ BAV and *Dcbld2*^−/−^ BAV treated with LDN-193189. ∗∗∗*P <* 0.001 (Mann-Whitney *U* test). Abbreviations as in Figures 1 and 3.

In the next set of studies, we sought to investigate whether the observed effects of DCBLD2 deficiency on VIC calcification in vitro are operational in vivo. Evaluation of *Bmp2* expression in aortic valves of WT and *Dcbld2*^−/−^ mice by RT-PCR demonstrated significantly higher *Bmp2* expression in *Dcbld2*^−/−^ compared with WT aortic valves (*P <* 0.01) (Figure 5A), whereas no significant difference could be detected between *Dcbld2*^−/−^ TAVs and BAVs (Supplemental Figure 6A). In stark contrast with this similar level of *Bmp2* expression between *Dcbld2*^−/−^ TAVs and BAVs, immunostaining showed significantly higher nuclear phosphorylated (p)SMAD1/5/9, an indicator of BMP2 signaling, in *Dcbld2*^−/−^ BAV compared with both WT and *Dcbld2*^−/−^ TAV leaflets (*P <* 0.001) (Figures 5B and 5C). Assessment of aortic valve expression of *Bmpr1a* and *Bmpr2*, major receptors that mediate BMP2 signaling,^6^ showed no difference between bicuspid and tricuspid *Dcbld2*^−/−^ (as well as WT) valves (Supplemental Figures 6B and 6C). Finally, we assessed aortic valve osteocalcin expression as a down-stream mediator of BMP2 signaling and marker of valvular calcification in CAVD.^6^^,^^20^, ^21^, ^22^ Like SMAD1/5/9 activation, osteocalcin expression was significantly higher in *Dcbld2*^−/−^ BAV leaflets compared with both WT (*P <* 0.05) and *Dcbld2*^−/−^ TAV (*P <* 0.01) leaflets (Figure 5D). Combined, these data suggest that *Bmp2* expression by itself is insufficient, and additional factors present to a higher extent in BAV are required to promote aortic valve BMP2 signaling and calcification.

### BMP inhibition and valvular calcification in BAV

Next, we sought to address to what extent this activation of BMP signaling is responsible for enhanced valvular calcification in BAV. As a prelude to in vivo studies, we assessed the effect of LDN-193189, a type 1 BMP receptor inhibitor,^23^ on SMAD1/5/9 phosphorylation in pVIC. LDN-193189 suppressed SMAD1/5/9 phosphorylation in both serum-exposed control cells (*P <* 0.05) and following siRNA-mediated DCBLD2 down-regulation (*P <* 0.001), which significantly enhances SMAD1/5/9 phosphorylation (*P <* 0.001) (Figures 5E and 5F). Next, a group of 1-year-old *Dcbld2*^−/−^ mice were treated with LDN-193189 (6 mg/kg/day, intraperitoneal) for 1 week, and the effect of the inhibitor on BMP signaling was evaluated by immunostaining and RT-PCR. On postmortem tissue analysis, LDN-193189 significantly reduced aortic valve SMAD1/5/9 phosphorylation (*P <* 0.001) (Figures 5G and 5H) and osteocalcin expression (*P <* 0.001) (Figure 5I) in animals with BAV to levels seen in WT and *Dcbld2*^−/−^ mice with TAV (Figure 5D). In animals with TAV, LDN-193189 had no effect on low levels of osteocalcin observed in nontreated animals (Supplemental Figure 7). Combined, these data indicate that BMP signaling is enhanced in valvular calcification of animals with BAV.

## Discussion

We established that DCBLD2 is down-regulated in human CAVD and identified a high incidence of BAV in *Dcbld2*^*−/−*^ mice. Taking advantage of this high incidence, here we show that 1) DCBLD2 deficiency promotes the development of CAVD and aortic stenosis, which is more severe in animals with BAV, 2) the difference between *Dcbld2*^*−/−*^ bicuspid and tricuspid valves is especially striking with regard to valvular calcification, which like in humans, affects the leaflets in these animals, and 3) BMP signaling is enhanced in BAV, despite a similarly up-regulated *Bmp2* expression in *Dcbld2*^*−/−*^ TAV and BAV. Combined, these findings indicate that in the context of the same genetic background, the presence of BAV promotes aortic valve calcification and stenosis through enhanced BMP2 signaling.

Valvular thickening, calcification, and aortic stenosis are key features of human CAVD. In humans, aortic valve calcification generally starts in the hinge region and may spare the aorta. These features are missing in many preclinical models of CAVD that are, for instance, based on hyperlipidemic animals, where valvular calcification is either absent or predominantly involves the annulus with some involvement of the leaflets, or Klotho deficiency, where the leaflets are not thickened and the animals do not develop aortic stenosis.^24^^,^^25^ This is in contrast to CAVD in *Dcbld2*^*−/−*^ mice that closely phenocopies the human disease. Accordingly, this is a potentially powerful model for preclinical studies of CAVD, which allowed us to define the interplay between leaflet numbers and genetic background. Similar to what happens in humans, a subset of TAV *Dcbld2*^*−/*−^ mice acquires aortic stenosis, and leaflet fibrosis, calcification, and stenosis are more prevalent in BAV animals.

DCBLD2 is a member of the DCBLD family of transmembrane proteins.^26^ Along with its less characterized homolog, DCBLD1, the 715–amino acid DCBLD2 has a structure consisting of a signal sequence, followed by CUB, LCCL, and coagulation factor V/VIII type-C (also discoidin) domains. This resembles the domain structure of neuropilins, which possess 2 CUB and discoidin domains. Recent studies have implicated DCBLD2 in the regulation of growth factor signaling. Accordingly, DCBLD2 deficiency promotes platelet-derived growth factor and insulin signaling in vascular smooth muscle cells, and inhibits vascular endothelial growth factor signaling in EC.^8^^,^^10^^,^^12^^,^^13^ In addition, DCBLD2 regulates epidermal growth factor–induced tumorigenesis.^11^ Our study extends the scope of those observations to demonstrate that in addition to regulating growth factor signaling in vascular and cancer cells, DCBLD2 modulates BMP2 expression and signaling in EC and VIC. This maybe through regulation of BMP receptor expression or phosphorylation, as shown previously for a number of growth factor receptors.^10^^,^^12^^,^^13^

BMP2 is a key osteogenic differentiation factor in vascular and valvular calcification.^6^^,^^27^ Canonical BMP2 signaling involves the binding of the ligand to a heterotetrameric complex of transmembrane type I and II serine/threonine kinase receptors, which triggers SMAD 1/5/9 phosphorylation and nuclear translocation to regulate gene expression.^28^ Previous in vitro studies had suggested that BMP signaling is necessary, but not sufficient, for VIC calcification, which requires a pro-osteogenic milieu.^6^ Our study confirmed the requirement for BMP signaling in VIC calcification in vitro, as Noggin, a BMP-specific inhibitor,^18^ attenuated VIC calcification in osteogenic medium. Similarly, the inhibitory effect of the BMP receptor inhibitor, LDN-193189, on osteocalcin expression in *Dcbld2*^*−/−*^ BAV established the critical role of BMP signaling in valvular calcification in vivo. Importantly, our results indicate that leaflet numbers modulate aortic valve BMP signaling, as evidenced by the higher SMAD1/5/9 phosphorylation and osteocalcin expression in bicuspid *Dcbld2*^*−/−*^ valves. This enhanced BMP signaling in conjunction with the modulatory effect of BMP inhibition on valvular calcification support the concept that like in vitro observations,^6^ BMP is required, but not sufficient, for valvular calcification in vivo. The contrast between the BAV and TAV flow patterns is well-recognized, with higher shear stresses, turbulence and unsteadiness found in models of BAV.^29^^,^^30^ These differences may underlie the observed differences in valvular calcification and BMP signaling between tricuspid and bicuspid valves in *Dcbld2*^*−/−*^ mice.

EC–VIC cross talk is implicated in maintaining the normal valve homoeostasis, as well as the development of CAVD.^19^^,^^31^^,^^32^ DCBLD2 deficiency transforms ECs from an anticalcific to a procalcific phenotype by promoting BMP2 production. However, endothelial DCBLD2 deficiency by itself is not sufficient for inducing CAVD in vivo. Although this may be related to the absence of BAV in these animals, it is also possible that concomitant DCBLD2 down-regulation in VICs is necessary to induce aortic valve calcification and stenosis.

Gene expression analyses of human BAV and TAV have identified several differentially expressed genes, such as insulin-like growth factor 1 and R-spondin 2, which is implicated in BMP-mediated mineralization.^33^ Although several genes, such as natriuretic peptide receptor 2 (*Npr2*)^34^ have been linked to BAV in mice, the genetic basis for the majority of human BAV cases remains undetermined.^19^^,^^35^^,^^36^ Notably, not every animal model of BAV is associated with CAVD, indicating that a combination of a predisposing genetic background and BAV is necessary for leaflet calcification and hemodynamically significant aortic stenosis to develop. In this regard, there is a paucity of information on the relation between the genetic background and CAVD. Notable exceptions are a single nucleotide polymorphism variant in the lipoprotein(a) locus that has been linked to aortic valve calcification in humans^37^ and the observation that the family members of BAV patients with NOTCH1 sequence variation who have a TAV are at higher risk for calcification.^4^^,^^5^ The development of CAVD in a subset of tricuspid *Dcbld2*^*−/−*^ mice and its severity in BAV caused by enhanced BMP signaling are directly relevant to the latter observation. The reduction in DCBLD2 expression in human CAVD and its regulation of BMP signaling directly link DCBLD2 to valvular calcification in humans. This also suggests that along with BMP2, DCBLD2 may be a therapeutic target to prevent CAVD progression, regardless of leaflet numbers.

### Study limitations

Although this does not affect the accuracy of the main conclusions of the study, we relied on osteocalcin as a dynamic surrogate marker of the calcification process in vivo, because it is highly sensitive to relatively short-term changes, and the detection of a reduction in calcium deposition in the valve requires long-term therapeutic interventions in numerous animals. Although DCBLD2 expression is reduced in human CAVD, the genetic basis of the majority of CAVD and BAV cases remains to be determined, and to date, linkage analyses have not identified any DCBLD2 sequence variation in human BAV. Finally, this study is focused on introducing a new preclinical model of BAV and CAVD that phenocopies human disease and the interplay between genetic background and leaflet numbers in promoting valvular calcification. The molecular mechanisms of BAV development in *Dcbld2*^*−/−*^ mice and regulation of BMP2 expression by DCBLD2 are beyond the scope of this first report and will be the subject of future studies.

## Conclusions

DCBLD2 expression is decreased in human CAVD, and a combination of genetic background, i.e., DCBLD2 deficiency, and BAV promotes aortic valve calcification and stenosis through enhanced BMP2 signaling in *Dcbld2*^*−/−*^ mice. This pathway may be a therapeutic target to prevent CAVD progression in BAV.

Perspectives **COMPETENCY IN MEDICAL KNOWLEDGE:** Patients with bicuspid aortic valve are at risk for accelerated, severe valvular calcification. Introducing a new murine model of bicuspid aortic valve that phenocopies human disease, we link this exaggerated calcification to enhanced bone morphogenic protein signaling despite similar levels of the protein in bicuspid compared with tricuspid aortic valve.

**TRANSLATIONAL OUTLOOK 1:** The *Dcbld2*^*−/−*^ mouse is a clinically relevant model to study the pathophysiology of CAVD. The absence of appropriate animal models of CAVD makes the *Dcbld2*^*−/−*^ model a powerful tool to study the pathophysiology of CAVD. The *Dcbld2*^*−/−*^ model also provides the opportunity to develop innovative therapies for CAVD, a disease for which there is no effective medical therapy.

**TRANSLATIONAL OUTLOOK 2:** Our data support the notion that BMP2 inhibition is a therapeutic target in CAVD, and modulation of DCBLD2 may be a novel pathway to prevent CAVD.

## Funding Support and Author Disclosures

This work was supported by grants from the National Institutes of Health (NIH) grants R01AG065917 and R01HL138567. and Department of Veterans Affairs grant I0-BX004038. Dr Salarian was supported by National Institutes of Health training grant 5T32HL007950. The resources of the Yale Translational Research Imaging Center, funded in part by National Institutes of Health grant 1S10RR018039, were used for this study. The authors have reported that they have no relationships relevant to the contents of this paper to disclose.

## Appendix

Supplemental Data

Supplemental Video 1 Example of a *Dcbld2*-/- murine tricuspid aortic valve.

Supplemental Video 2 Example of a *Dcbld2*-/- murine bicuspid aortic valve.
