# Supplementary material for: Differential BMP Signaling Mediates the Interplay Between Genetics and Leaflet Numbers in Aortic Valve Calcification
Source: JACC Basic Transl Sci. 2022 Mar 23;7(4):333–45. doi: 10.1016/j.jacbts.2021.12.006 (PMC9079798; doi:10.1016/j.jacbts.2021.12.006)
Supplement: Supplemental Data [file mmc1.pdf]

Online Material

Differential BMP signaling mediates the interplay between genetics and leaflet numbers in aortic valve calcification

Brief title: BMP signaling and BAV calcification

Jae-Joon Jung, PhD[a,b#], Azmi A. Ahmad, PhD[a,b#], Saranya Rajendran, PhD[a,b], Linyan Wei, MD[a,b], Jiasheng Zhang, MD[a,b], Jakub Toczek, PhD[a,b], Lei Nie, PhD[a,b,†], Gunjan Kukreja, PhD[a,b], Mani Salarian, PhD[a,b], Kiran Gona, PhD[a,b], Mean Ghim, PhD[a,b], Raja Chakraborty, PhD[a], Kathleen A. Martin, PhD[a], George Tellides, MD, PhD[b,c], Donald Heistad, MD[d], Mehran M. Sadeghi, MD[a,b,*]

[a] Section of Cardiovascular Medicine and Cardiovascular Research Center, Yale University School of Medicine, New Haven, CT, United States.

[b] VA Connecticut Healthcare System, West Haven, CT, United States.

[c] Department of Surgery, Yale University School of Medicine, New Haven, CT, United States.

[d] Division of Cardiovascular Medicine, University of Iowa Carver College of Medicine, Iowa City, IA, United States.

# Equal contribution

*To whom correspondence should be addressed

Detailed Material and Methods

Reagents. The polyclonal rabbit anti-DCBLD2 antibody was purchased from Sigma-Aldrich (St. Louis, MO). The antibody for BMP2 was obtained from Abcam (Cambridge, MA). The antibodies for phospho-SMAD1/5/9, SMAD1, and glyceraldehyde 3-phosphate dehydrogenase (GAPDH) were obtained from Cell Signaling Technologies (Danvers, MA). BMP2 (against a c-terminal immunogen for the secreted form) and COL1A1 antibodies were purchased from Novus Biologicals (Centennial, CO). The anti-CD31 antibody was obtained from BD Biosciences (San Jose, CA). Other reagents were purchased from Sigma-Aldrich, unless stated otherwise.

Human tissues. Normal human aortic valves were obtained from deceased organ donors, and aortic valves with advanced CAVD were obtained from anonymous patients undergoing aortic valve replacement for symptomatic aortic stenosis under protocols approved by Yale Institutional Review Board.

Animal models. The generation of *Dcbld2*$^{-/-}$ mice on C57BL/6 background and endothelial-specific conditional knockout mice (*Cdh5-Cre*/*Dcbld2*$^{fl/-}$) were reported previously (1). Both sexes are used in these studies and to simplify, the data from 9-12 months old mice are grouped together. Aortic valves harvested under isoflurane anesthesia were frozen immediately in OCT media until further analysis. For LDN-193189 treatment, 1-year old *Dcbld2*$^{-/-}$ mice were randomly divided into sex-matched control and treatment groups. The treatment group received 6 mg/kg LDN-193189 daily for 1 week via intraperitoneal injection. Distilled water was administered for the control group. All animal procedures were performed in accordance with protocols approved by Yale University and Veterans Affairs Connecticut Healthcare System Institutional Animal Care and Use Committees.

Echocardiography. M-mode and 2D echocardiographic images were obtained using a VisualSonics Vevo 2100 system with a 30MHZ probe. The spatial resolution and M-mode frame rate of the system are respectively, 30 µm and ~1000/second. The animals were anesthetized with 1-2% isoflurane in 100% oxygen and body temperature was monitored using a rectal probe. Repeated M mode measurements were performed to measure minimal systolic aortic valve leaflet separation, LV ejection fraction, and body surface area-corrected LV mass. Flow velocities were measured with pulsed wave Doppler.

Histology, Morphometry and Immunostaining. Hematoxylin and eosin (H&E) and Masson's trichrome staining were performed on five µm thick sections by Yale Pathology Tissue Services using standard techniques. Aortic valve leaflet thickness was measured on H&E-stained sections imaged with a light microscope equipped with a Leica MC 170 HD camera (Leica, Wetzlar, Germany). Leaflet thickness was measured at regular intervals on all leaflets using fiji/Image J software and averaged. Other sections were fixed with 4% paraformaldehyde in PBS for 20 min at room temperature followed by blocking with 10% normal goat serum in PBS for 1 hour. For immunofluorescence, the sections were incubated with primary antibodies overnight at 4°C. The sections were then incubated for 1 hour with either Alexa Fluor 488- or Alexa Fluor 594-conjugated secondary antibodies (Thermo Fisher Scientific, Waltham, MA) followed by DAPI staining (Thermo Fisher Scientific). After washing, the slides were mounted using Prolong Diamond Antifade Mountant (Thermo Fisher Scientific). Fluorescence images were acquired using a Nikon AR1 confocal microscope and quantified using Image J software. For immunohistochemistry, tissue sections were incubated with primary antibodies for 1 hour at room temperature. The sections were then incubated with biotinylated secondary antibodies in 1:200 dilution for 1 hour followed by incubation in Avidin-Biotin Complex Kits (Vector

Laboratories, Burlingame, CA) for 30 min, exposure to 3-3' diaminobenzidine substrate, and counter staining with hematoxylin.

Cell culture. Hearts from 3-month old male Yorkshire pigs were provided by Yale Animal Resources Center within 1 hour of euthanasia. Primary porcine valvular endothelial cells (pVEC) were harvested from porcine aortic valve leaflets by digesting the endothelial layer with 2 mg/ml of collagenase for 30 minutes. The leaflets were then cleaned and incubated with 0.8 mg/ml of collagenase overnight to obtain porcine valvular interstitial cells (pVIC). pVEC were plated and cultured on a 0.5% gelatin-coated culture flask in medium 199 containing 20% fetal bovine serum (FBS), glutamine, penicillin-streptomycin, and endothelial cell growth supplement, and identified as endothelial cells by flow cytometry for CD31. pVIC were plated and cultured on a culture flask in Dulbecco's modified eagle medium containing 10% FBS and 1% penicillin-streptomycin. Experiments were performed with three independent batches of cells pooled together, unless stated otherwise. Cells were used between passages 4 to 6. Human aortic valve VIC (hVIC) were isolated from transplant donors using the pVIC isolation protocol. Stable DCBLD2-deficient hVIC were generated using lentiviral shRNA (Santa Cruz Biotechnology) and puromycin selection. Lentiviral particles expressing scrambled shRNA were used to generate control cells. Mouse lung endothelial cells (MLEC) were isolated from 4-week-old wild type (WT) and *Dcbld2*$^{-/-}$ mice by two rounds of immunoselection with anti-CD31- and ICAM-2-conjugated magnetic beads and their purity was confirmed by flow cytometry, as described (1,2). For BMP2 signaling assays, porcine valvular cells were transfected with siRNAs (scrambled or porcine *DCBLD2*, sequence: GAACUUGUUUCAGAUGGCUGAAAUA, Thermo Fisher Scientific, Waltham, MA) for 48 hours. Following culture in serum-reduced medium (1% FBS) for 24 hours, they were treated with BMP2 (50 ng/ml) for the indicated times. For in vitro

calcification assays, 3.0 x 10^5 pVIC were seeded in each well of 12-well plate. One day after seeding, the cells were transfected with siRNAs for 24 hours, and then the culture media was replaced with osteogenic differentiation media (Lonza, Basel, Switzerland) for 72 hours with or without human recombinant Noggin (500 ng/ml). The cells were then washed and fixed with 4% paraformaldehyde. In brief, the fixed cells were incubated in 2% Alizarin red S solution for 5 minutes. After three washes with PBS, the cells were dried before imaging with a Leica MZ9.5 stereomicroscope. Alizarin-red positive calcific nodules were counted using Fiji/Image J software. For LDN-193189 assays, pVIC were transfected with siRNA with or without 0.5 µM LDN-193189 for 48 hours. MLEC-pVIC co-culture was performed using transwell inserts (Corning, Lowell, MA). 1.0 x 10^6 pVIC were seeded in the bottom chamber (6-well plate) and 0.2 x 10^6 MLEC were seeded in the upper chamber (24 mm diameter, 0.4 µm pore size). Cells were cultured for 11 days and the number of calcified nodules in the bottom chambers were counted. Experiments were performed in triplicate.

Western blotting. Western blotting was performed on protein extracts as described with minor modification (1). Proteins were extracted in ice-cold RIPA buffer (150 mM NaCl, 50 mM Tris·HCl, pH 8.0, 1.0% Nonidet P-40, 0.5% sodium deoxycholate, and 0.1% SDS) supplemented with complete proteinase inhibitor (Roche Applied Sciences, Indianapolis, IN) and phosphatase inhibitor cocktail (Sigma-Aldrich). For phosphorylated antibodies, the membranes were stripped by restore plus Western blot strip buffer (Thermo Fisher Scientific, Rockford, IL) and re-probed with total protein antibodies. GAPDH was used as loading control. To quantify secreted BMP2, culture media were replaced with FBS deficient media after 48 hours of siRNA transfection. The conditioned media were collected after 24 hours, centrifuged and equal volumes of the supernatant were used for Western blotting. GAPDH expression in the same volume of protein

extracts from the remaining adherent cells served as the loading control. The blots were imaged using ChemiDoc MP Imager systems (Bio-Rad Laboratories, Hercules, CA), and quantified using Image J software.

Real time-PCR. Total RNA was isolated and reverse transcribed using QIAGEN kits (Valencia, CA). RT-PCR was performed in triplicates using TaqMan gene assays (Thermo Fisher Scientific) for human and mouse samples and QuantiTect SYBR Green PCR Kits (QIAGEN) for porcine samples according to manufacturers' instructions. The data for human and mouse samples were normalized to 18S rRNA and the results of porcine samples were normalized to GAPDH. The following Taqman primers, 4352930E, Hs00294635_m1, Mm00472304_m1, Mm01340178_m1, Mm00477650_m1, Mm00432134_m1, Mm03413826_mH were used for eukaryotic 18s rRNA, human *DCBLD2*, mouse *Dcbld2*, mouse *Bmp2*, mouse *Bmpr1a*, mouse *Bmpr2*, and mouse Osteocalcin, respectively. For porcine gene, the following primers were designed for SYBR Green PCR: forward ATGATTCCACCCACGGCAAG, reverse AGAAGGGGCAGAGATGATGAC for porcine *GAPDH*, forward CCTGCTTTAGTGGGAACATACA, reverse CGGGCTGAAGAGCAGCTA for porcine *DCBLD2*, forward GACCTCATTCCCGAGCTG, reverse GGAAGAGGAGAGGCCAGTAGA for porcine *BMP2*.

Supplemental Figures

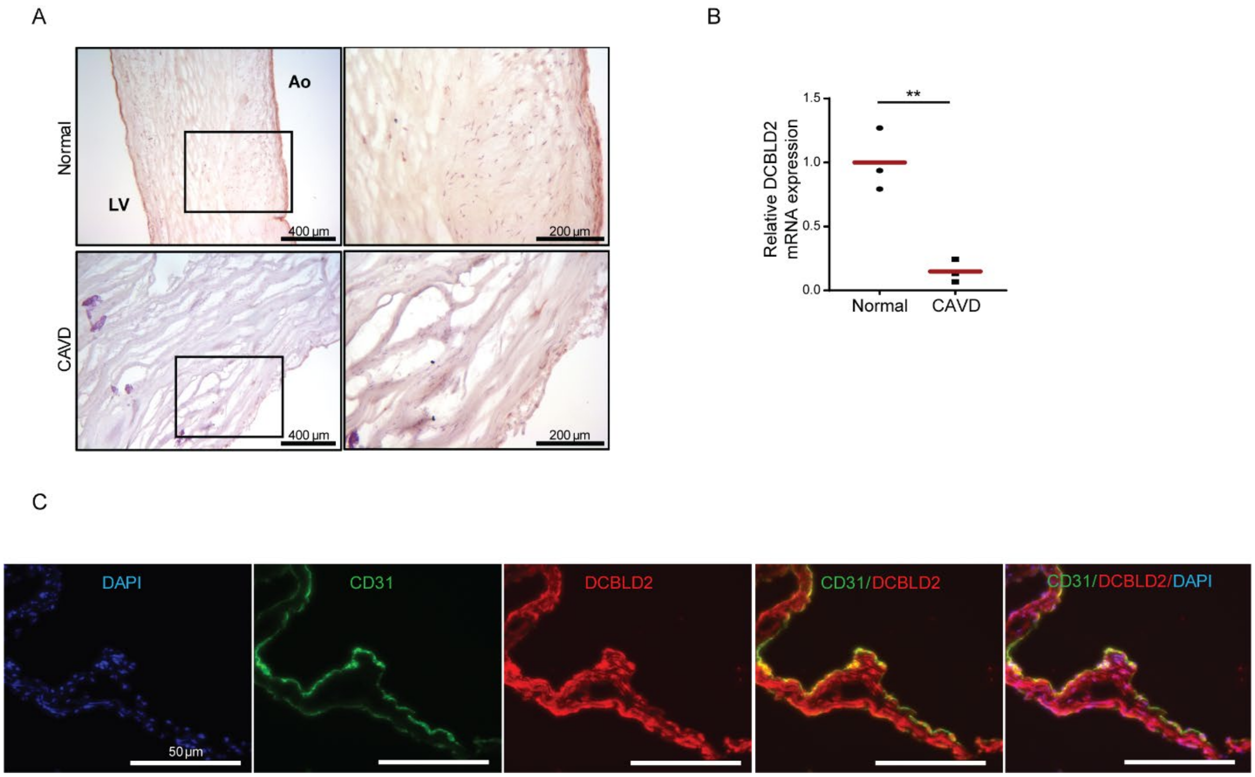

Supplemental Figure 1. DCBLD2 expression in human and murine aortic valves. A: Immunohistochemical staining of DCBLD2 in normal human aortic valve leaflets (upper panels) and aortic valve leaflets with advanced CAVD (lower panels). Ao: aorta. LV: left ventricle. B: *DCBLD2* mRNA expression in normal human aortic valve leaflets (upper panels) and aortic valve leaflets with advanced CAVD. **: $P < 0.01$ (two-tailed t-test). C: DCBLD2 (in red) and CD31 (in green) expression in a wild type mouse aortic valve leaflet. Nuclei are stained blue with DAPI. Scale bar: 50 µm.

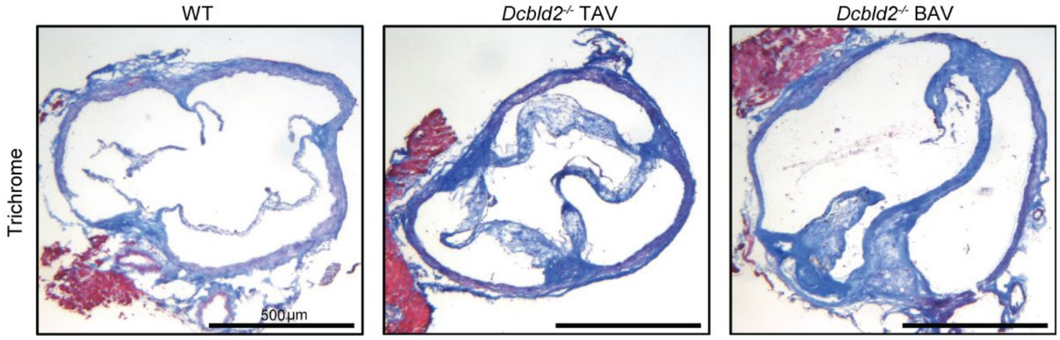

Supplemental Figure 2. Aortic valve fibrosis in *Dcbld2*$^{-/-}$ mice. Masson's trichrome staining of murine WT, *Dcbld2*$^{-/-}$ tricuspid, and *Dcbld2*$^{-/-}$ bicuspid aortic valves. Scale bar: 500 µm.

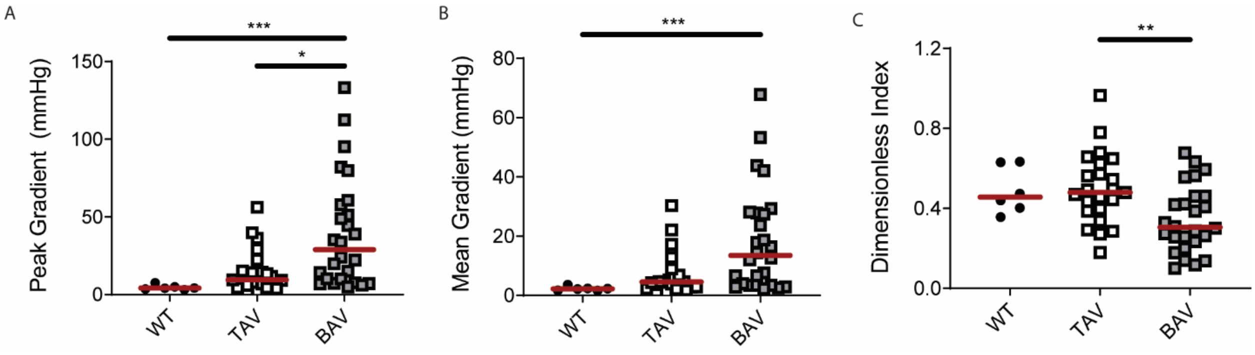

Supplemental Figure 3. Aortic valve physiology in *Dcbld2*$^{-/-}$ mice. Aortic valve peak gradient (A), mean gradient (B), and dimensionless index (C) assessed by Doppler echocardiography in WT, *Dcbld2*$^{-/-}$ TAV and *Dcbld2*$^{-/-}$ BAV mice. *: $P < 0.05$, ***: $P < 0.001$ (Kruskal-Wallis test with Dunn's multiple comparison), **: $P < 0.01$ (One-way ANOVA with Tukey's multiple comparison).

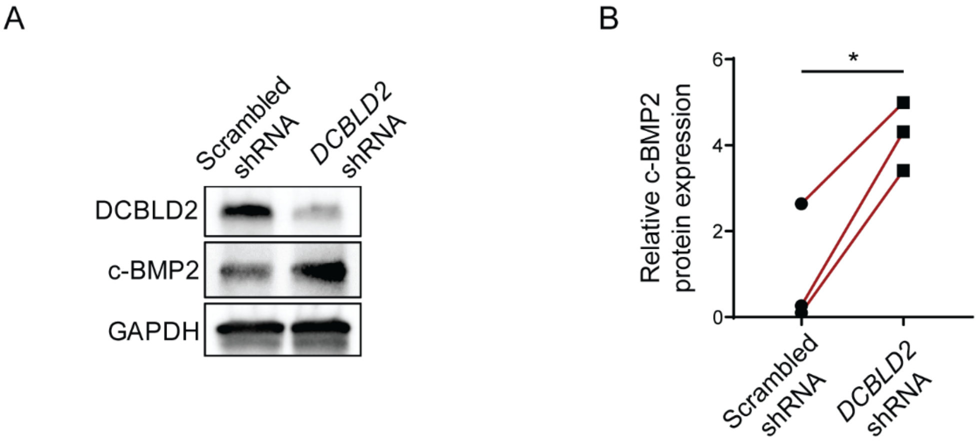

Supplemental Fig. 4. shRNA-mediated DCBLD2 downregulation promotes BMP2 expression in human valvular interstitial cells**.** Representative Western blot (A) and quantification of cytosolic BMP2 protein expression in human valvular interstitial cells. *: $P < 0.05$ (paired t-test).

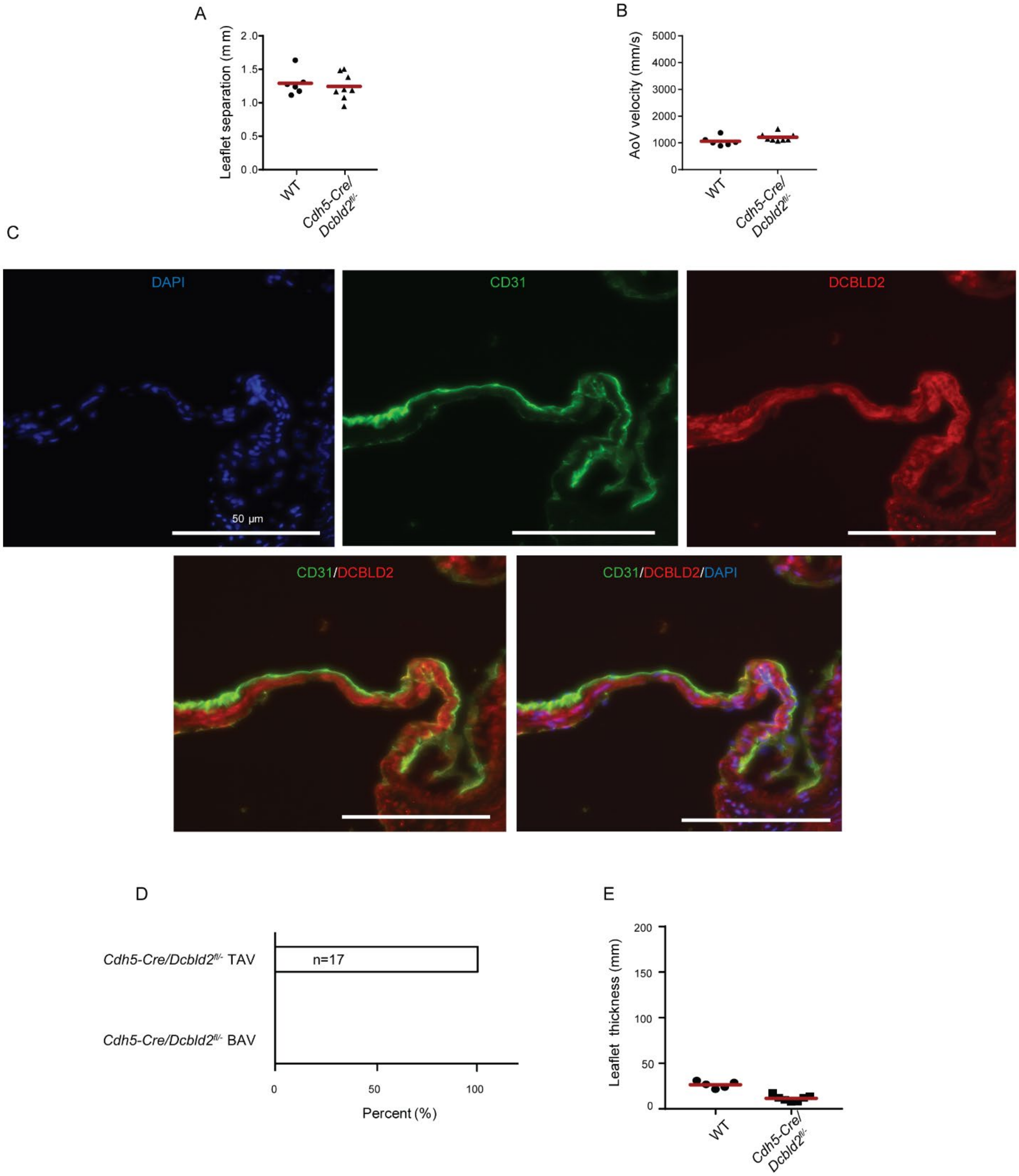

Supplemental Figure 5. Effect of endothelial cell *Dcbld2* deletion on aortic valve structure and function. A, B: Aortic valve leaflet separation (A) and peak transaortic valve flow velocity (B) in WT and *Cdh5*-Cre/*Dcbld2*$^{fl/-}$ mice assessed by echocardiography. C: DCBLD2 (in red) and

CD31 (in green) expression in a *Cdh5*-Cre/*Dcbld2*$^{fl/-}$ mouse aortic valve leaflet. Nuclei are stained blue with DAPI. Scale bar: 50 µm. D: Prevalence of TAV and BAV in *Cdh5*-Cre/*Dcbld2*$^{fl/-}$ mice. E: Aortic valve leaflet thickness of WT and *Cdh5*-Cre/*Dcbld2*$^{fl/-}$ mice.

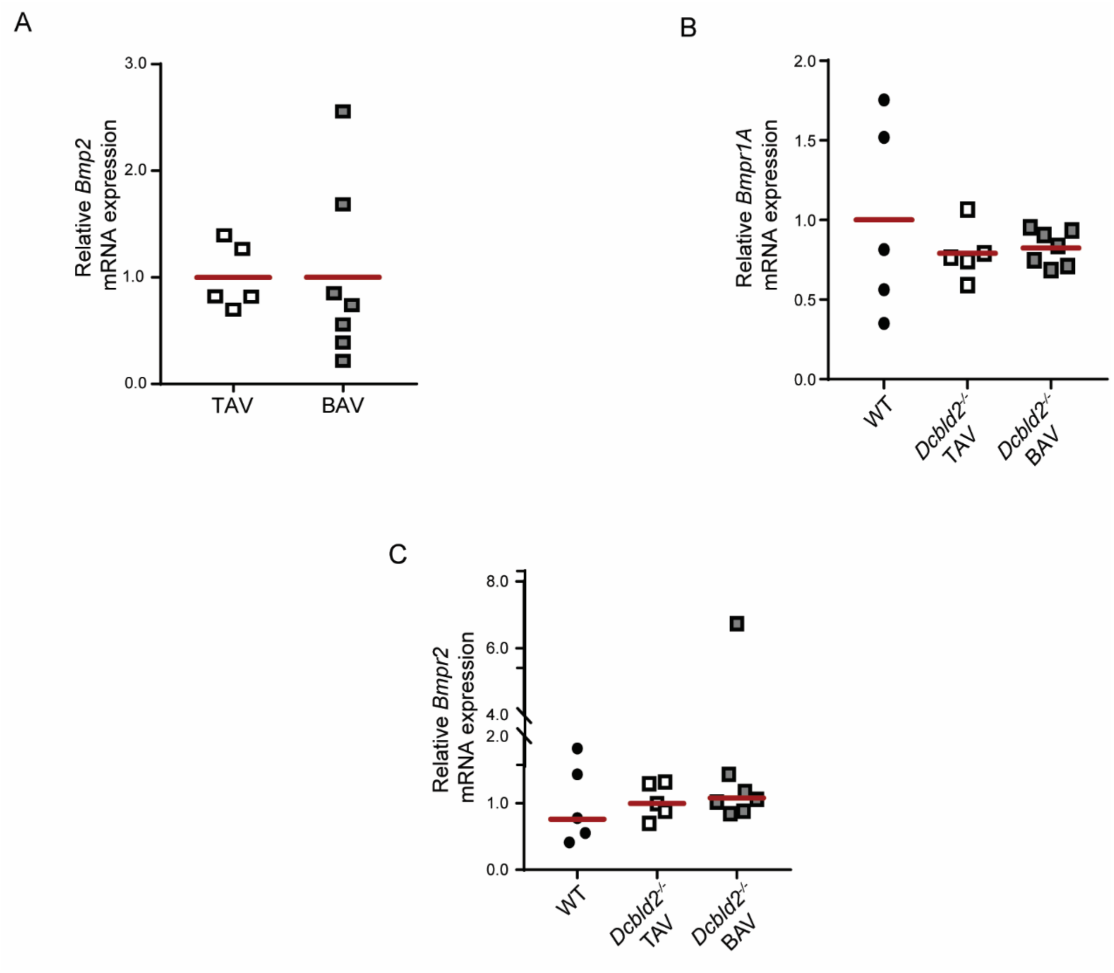

Supplemental Figure 6. *Bmp2* and BMP receptor expression in *Dcbld2*$^{-/-}$ mice. A**:** *Bmp2* mRNA expression in *Dcbld2*$^{-/-}$ TAV and *Dcbld2*$^{-/-}$ BAV (Welch's t-test). B, C: *Bmpr1a* (B) and *Bmpr2* (C) mRNA expression in WT aortic valves, *Dcbld2*$^{-/-}$ TAV and *Dcbld2*$^{-/-}$ BAV (one-way ANOVA with Tukey's multiple comparisons for *Bmpr1a*, Kruskal-Wallis test with Dunn's multiple comparisons for *Bmpr2*).

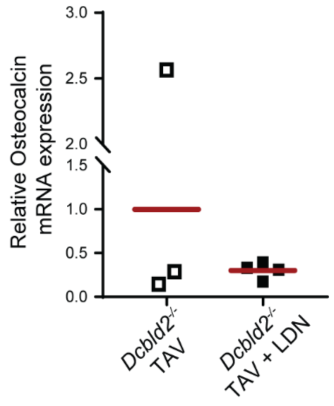

Supplemental Figure 7. Effect of LDN on Osteocalcin mRNA expression in *Dcbld2*$^{-/-}$ mice with tricuspid aortic valve (TAV, Two-tailed t-test).

Supplemental Video 1. Example of a *Dcbld2*$^{-/-}$ murine tricuspid aortic valve.

Supplemental Video 2. Example of a *Dcbld2*$^{-/-}$ murine bicuspid aortic valve.

References

1. Nie L, Guo X, Esmailzadeh L et al. Transmembrane protein ESDN promotes endothelial VEGF signaling and regulates angiogenesis. J Clin Invest 2013;123:5082-97.

2. Luo D, Luo Y, He Y et al. Differential functions of tumor necrosis factor receptor 1 and 2 signaling in ischemia-mediated arteriogenesis and angiogenesis. Am J Pathol 2006;169:1886-98.
